# Recalibrating the notion of modelling for policymaking during pandemics

**DOI:** 10.1016/j.epidem.2022.100552

**Published:** 2022-03

**Authors:** Yot Teerawattananon, Sarin KC, Y.-Ling Chi, Saudamini Dabak, Joseph Kazibwe, Hannah Clapham, Claudia Lopez Hernandez, Gabriel M. Leung, Hamid Sharifi, Mahlet Habtemariam, Mark Blecher, Sania Nishtar, Swarup Sarkar, David Wilson, Kalipso Chalkidou, Marelize Gorgens, Raymond Hutubessy, Suwit Wibulpolprasert

**Affiliations:** a Health Intervention and Technology Assessment Program (HITAP), Department of Health, Ministry of Public Health, 6th Floor, 6th Building, Tiwanon Road, Nonthaburi 11000, Thailand; b Centre for Global Development Europe, Great Peter House, Abbey Gardens, Great College St, Westminster, London SW1P 3SE, UK; c Department of Infectious Disease Epidemiology, School of Public Health, Imperial College London (ICL), Faculty of Medicine Building, St Mary’s Campus, Norfolk Place, London W2 1PG, UK; d Saw Swee Hock School of Public Health (SSHSPH), National University of Singapore (NUS), 12 Science Drive 2, #10-01, 117549, Singapore; e Mayor’s Office City of Bogota, Bogota City Hall, 111711, Colombia; f Li Ka Shing Faculty of Medicine (HKUMed), Hong Kong University, 21 Sassoon Rd, Pok Fu Lam, Hong Kong; g HIV/STI Surveillance Research Center, and WHO Collaborating Center for HIV Surveillance, Institute for Futures Studies in Health, Kerman University of Medical Sciences (KMU), Kerman 7616911320, Iran; h Africa Centres for Disease Control and Prevention, African Union Commission, Roosevelt Streeet, Addis Ababa, Ethiopia; i National Treasury, 120 Plein Street, Cape Town, Republic of South Africa; j Poverty Alleviation and Social Safety Division, Government of Pakistan, Cabinet Secretariat, 4th Floor, Evacuee Trust Complex, F-5/1, Islamabad, Pakistan; k Indian Council for Medical Research (ICMR), Government of India, V. Ramalingaswami Bhawan, P.O. Box No. 4911, Ansari Nagar, New Delhi 110029, India; l Bill and Melinda Gates Foundation (BMGF), 500 5th Ave N, Seattle, WA 98109, USA; m The Global Fund to Fight AIDS, Tuberculosis and Malaria, Global Health Campus, Chemin du Pommier 40, 1218 Grand-Saconnex, Geneva, Switzerland; n World Bank Group (WBG), 1818H Street, N.W., Washington, DC 20433, USA; o World Health Organisation (WHO), Avenue Appia 20, 1211 Geneva, Switzerland; p International Health Policy Program (IHPP), Ministry of Public Health, Tiwanon Rd., Nonthaburi 11000, Thailand

## Abstract

COVID-19 disease models have aided policymakers in low-and middle-income countries (LMICs) with many critical decisions. Many challenges remain surrounding their use, from inappropriate model selection and adoption, inadequate and untimely reporting of evidence, to the lack of iterative stakeholder engagement in policy formulation and deliberation. These issues can contribute to the misuse of models and hinder effective policy implementation. Without guidance on how to address such challenges, the true potential of such models may not be realised. The COVID-19 Multi-Model Comparison Collaboration (CMCC) was formed to address this gap. CMCC is a global collaboration between decision-makers from LMICs, modellers and researchers, and development partners. To understand the limitations of existing COVID-19 disease models (primarily from high income countries) and how they could be adequately support decision-making in LMICs, a desk review of modelling experience during the COVID-19 and past disease outbreaks, two online surveys, and regular online consultations were held among the collaborators. Three key recommendations from CMCC include: A ‘fitness-for-purpose’ flowchart, a tool that concurrently walks policymakers (or their advisors) and modellers through a model selection and development process. The flowchart is organised around the following: policy aims, modelling feasibility, model implementation, model reporting commitment. Holmdahl and Buckee (2020) A ‘reporting standards trajectory’, which includes three gradually increasing standard of reports, ‘minimum’, ‘acceptable’, and ‘ideal’, and seeks collaboration from funders, modellers, and decision-makers to enhance the quality of reports over time and accountability of researchers. Malla et al. (2018) A framework for “collaborative modelling for effective policy implementation and evaluation” which extends the definition of stakeholders to funders, ground-level implementers, public, and other researchers, and outlines how each can contribute to modelling. We advocate for standardisation of modelling processes and adoption of country-owned model through iterative stakeholder participation and discuss how they can enhance trust, accountability, and public ownership to decisions.

## Highlights

• COVID-19 models need appropriate adaptation to reflect contextual differences across settings.
• Upholding scientific standards is equally important as providing evidence for policymaking during pandemics.
• Wider stakeholder engagement with an iterative process for re-evaluating decisions is required for effective policy implementation.

## 1 Introduction

There is growing reliance on readily available COVID-19 disease models developed in high-income countries (HICs) to adapt to low and middle-income countries (LMICs). Lack of technical capacity to develop models locally and the demand for urgent decision-making may be contributing to this trend. While the need for local models is undebated (Caiado BCaC, 2020), the impact of such adapted models may be impeded due to challenges surrounding their use, from inappropriate model selection, inadequate and untimely reporting of evidence, and lack of iterative stakeholder engagement in policy formulation and implementation (Holmdahl and Buckee, 2020). Models built in HICs for HICs, by their sheer size, resources available, and number of people involved in their development, have had more opportunities to be validated, iterated, and contextualised. However, the same cannot be said for LMICs where effective knowledge transfer in public health are hindered by complexities in creating and accessing evidence, contextualising knowledge translations strategies, among others factors (Malla et al., 2018). Hence, the full set of locally relevant constraints may not be adequately reflected in these global models that are adapted for LMICs. Together, these elements can contribute to the misuse of models and hinder effective policy implementation. In the absence of guidance to identify and address such challenges, the true potential of such models may not be realised.

In response, we convened a Policy Group comprising decision-makers from LMICs who were engaged in responding to the pandemic in their respective countries under the COVID-19 Multi-Model Comparison Collaboration (CMCC). Members of this group represented the Ministry of Public Health, Thailand, Africa Center for Disease Control and Prevention (CDC), Mayor’s Office of the City of Bogota, Prime Minister’s Office of Pakistan, Indian Medical Research Council (ICMR), Government of India, National Treasury of South Africa, COVID-19 Task Force of Hong Kong, and Government of Iran. The CMCC is a collaboration between policymakers, disease modelling experts, and development partners, a global initiative led by a group of organisations representing different constituencies namely, the Bill and Melinda Gates Foundation (BMGF), the International Decision Support Initiative (iDSI), the World Bank Group (WBG), and the World Health Organisation (WHO). As part of this engagement, we organised consultations, conducted two online surveys, and a desk review of modelling experience during the COVID-19 and past disease outbreaks. The online surveys were conducted among decision-makers from LMICs, COVID-19 disease modellers and researchers, and international funders. The purpose of these surveys was to understand the limitations of existing COVID-19 disease models (primarily from HICs) and how they could be adequately adapted to LMICs. This engagement led to the development of our policy report, *‘Guidance on Use of Modelling for Policy Responses to COVID-19′* (COVID-19 Multi-Model Comparison Collaboration (CMCC) Policy Group, 2020), which intends to support decision-makers, their advisors, and the research community to build greater collaboration for effective policy implementation and evaluation. Our technical report, *‘Model Fitness-for-Purpose Assessment Report’* (COVID-19 Multi-Model Comparison Collaboration (CMCC) Technical Group Report, 2020), provides a comprehensive guide on model comparison, outlining their purpose, strengths and weaknesses, data needs, etc., and how they can be tailored to the local context. These reports are complimentary, therefore, best read in conjunction.

In this paper, we summarise key findings and recommendations from the report and discuss their implications on research and policy implementation. Throughout this paper, we advocate for the standardisation of modelling process and the adoption of country-owned models through stakeholder participation and discuss how they can enhance strength of evidence, trust, accountability, and public ownership to decisions. While our recommendations are tangible in nature, they should be viewed as frameworks with general principles to follow while modelling for decision making. We urge countries to contextualise our recommendations, depending on the resources available, policy problem, and stakeholders involved and their roles in the country, and draft individualised action plans as necessary. The strength of our approach lies in the global stakeholder engagement undertaken to understand the issues related to model use and resulting decision tools that were reviewed and endorsed as ‘necessary’. Applied to this outbreak, these tools can ensure that decision-makers and their advisors are equipped to adequately use such models and ensure they are fit-for-purpose for local decision-needs.

## 2 A framework to support informed use of models in decision-making

Many types of COVID-19 disease models exist with varying structure, assumptions, strengths, limitations, and abilities to address a given decision problem (COVID-19 Multi-Model Comparison Collaboration (CMCC) Technical Group Report, 2020). It is worth noting that these models are not designed to answer all COVID-19 related questions decision-makers may have (Layne et al., 2020). For instance, the issue of vaccine hesitancy cannot be understood using disease models. This requires estimating the magnitude (number of people) of vaccine hesitancy in the country and understanding their profile (their reasons for being hesitant). Such information may be retrieved via nation-wide surveys and not through disease models. Other examples include measuring real-world effectiveness of COVID-19 vaccines or efficacy of mask wearing in preventing infection which require other forms of research technique (for example, randomised controlled or observational studies using regression analysis), and therefore, not addressable by disease models. However, these are crucial pieces of information used by disease models to estimate parameters such as the $R_{0}$ and predict the outcomes of the pandemic such as number of cases, required hospital beds, etc. Disease models are therefore not a panacea for all policy questions, and it is important to distinguish questions not addressable by them and communicate to decision-makers who may need to resort to other research methods or groups for answers. In-depth discussion on the limitations of such models have been discussed elsewhere (Holmdahl and Buckee, 2020, COVID-19 Multi-Model Comparison Collaboration (CMCC) technical group report). Furthermore, one must recognise all models can be prone to inaccurate predictions, for example, consistent overestimation of COVID-19 deaths or resources required in hospitals during the early stages of the pandemic (Holmdahl and Buckee, 2020). However, models remain the best tool available to us to project future outcomes and draft appropriate response. A large part of model inaccuracies may be attributed to poor quality and limited data or model structure not capturing the extent of disease dynamics and local setting, especially when dealing with a novel virus. Hence, predictions must be accompanied by uncertainties and their implications on whether reasonable decisions can still be made. This can be used by decision-makers when communicating the level of confidence they have on a certain policy based on model findings. For those reasons, blindly adopting a model without understanding its purpose and limitations can be costly in lives, time, and resources wasted, and must be avoided. Hence, there is a need for guidance that concurrently walks decision-makers and modellers in the approach for fine tuning the model selection and development process.

**Fig. 1:**
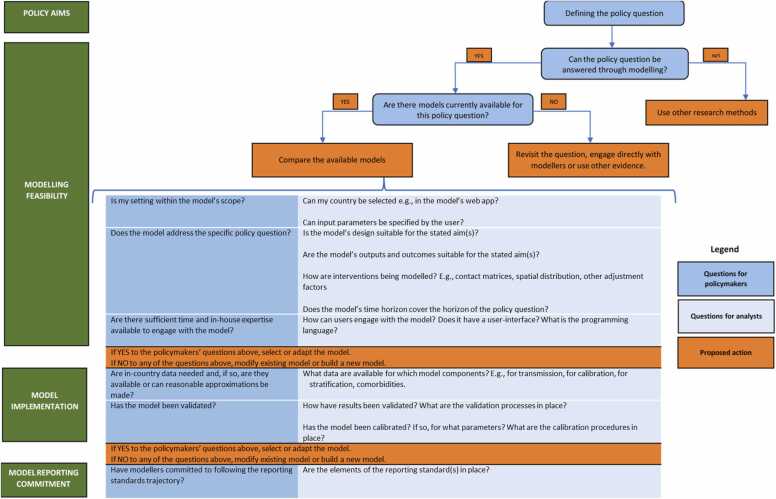
Fitness-for-purpose flowchart.

Catering to this need, we designed a fitness-for purpose flowchart, depicted in Fig. 1. The flowchart is a model selection and development tool which is organised around the following: policy aims, modelling feasibility, model implementation, model reporting and commitment. The aim is to guide policymakers through a sequence of questions that should be raised while considering the use of models without discussing in detail about technical issues, for instance, each data needed to populate the model and how to compare, assess, calibrate, or validate models. Comprehensive guidance on these issues is available in Tables 2–6 of our technical report (COVID-19 Multi-Model Comparison Collaboration (CMCC) Technical Group Report, 2020). The flowchart helps assess the suitability of models to answer a given policy question, availability of appropriate models, and feasibility in implementing such models to a local setting. The flowchart ends with questions around reporting standards (discussed in the subsequent section).

The use of this flowchart is a collaborative process that offers benefits beyond just the selection of the right model. Direct communication with policymakers or their advisors means the modellers can identify whether their modelling efforts are serving the decision needs of the hour. If not, modelling efforts can be repurposed. Decision-makers can further contribute to this process by bringing insights on policymaking and country context (political or wider context), as well as collaborations that can support data collection, model calibration, and validation where appropriate. Modellers can help shape decision-makers’ perspectives on the decision-problem and viable interventions by pointing to relevant literature or international experience. This two-way communication can help identify gaps in the current modelling effort for e.g., lack of local data, in-house technical capacity to produce, appraise, and consume evidence, etc. which the funders, decision-makers, and modellers can collectively find means to invest in and improve on. This can help mitigate issues resulting from inaccurate predictions from models or finding other methods to address policy questions as highlighted earlier. We discuss this further in our collaborating modelling framework.

Not all factors highlighted in the flowchart can always be fully met, therefore, modellers and decision-makers need to balance the trade-offs and decide where to compromise. The extent to which a model is fit-for-purpose will ultimately be a matter of judgement resulting from an ongoing dialogue between policymakers and modellers, informed by the answers to these questions. Hence, we encourage an iterative model development and selection process to ensure evolving disease dynamics, newly available evidence, and evolving decision problems are adequately captured and addressed by models. This flowchart has been applied during the pandemic and was adopted in Singapore and Thailand while modelling the impact and conducting economic evaluations of several COVID-19 vaccines which subsequently informed the national vaccination policy (Painter et al., 2021)*.*

## 3 Upholding scientific standards and accountability

“Crises are no excuse for lowering scientific standards”, say ethicists from Carnegie Mellon and McGill Universitie (Carnegie Mellon University, 2020). Since the outbreak, the speed at which evidence is being generated is unparalleled and maintaining the quality of evidence and accountability of evidence producers and users have been problematic (Boseley HDaS, 2020, Retraction Watch, 2020). Decisions based on inappropriate, inadequate, and untimely evidence can have lasting health and economic repercussions (Baral et al., 2020). The absence of internationally accepted guidelines to address such issues warrants a framework for upholding scientific standards and accountability of stakeholders during unprecedented times.

**Fig. 2:**
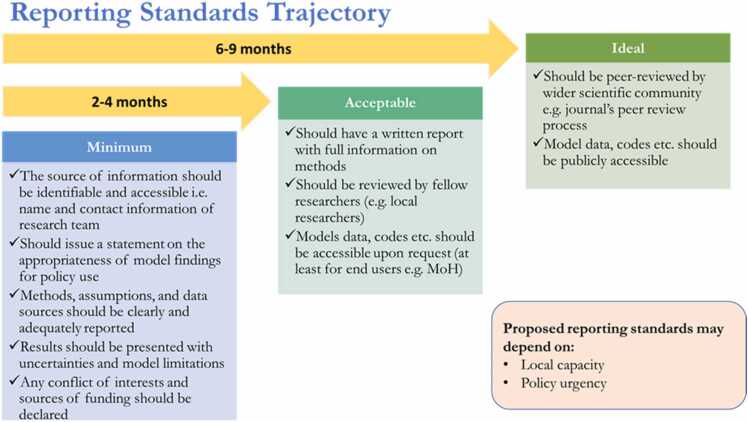
Reporting standards trajectory.

To this aim, we propose a ‘Reporting Standards Trajectory’ (RST) comprising three gradually increasing standard of reports, ‘minimum’, ‘acceptable’, and ‘ideal’, that seek collaboration from funders, modellers, and decision-makers to enhance the quality of reporting model findings over time. As shown in Fig. 2, each stage of reporting is bound by a set of criteria that are guided by key principles that govern a high quality of evidence from models (Wilkinson et al., 2016): Translation of a policy question to a research question, selection of an appropriate model, contextualisation of the model, model validation, incorporating uncertainties, and declaration of conflict of interest. Furthermore, modellers are asked to report a preferred mode of contact, which provides decision-makers a means to communicate with modellers for clarification or future iteration, make their models (codes, data, etc.) publicly accessible, get their work peer-reviewed for public scrutiny and gain validation from the wider scientific community. This is particularly relevant given the recent evidence of poor quality and low publication rate (ranging from 6% to 21%) in peer-reviewed journals among pre-printed manuscripts related to COVID-19 (Anazco et al., 2021). This suggests that failures in peer review may also constitute a public health risk if the results of non-reviewed studies were used to inform policy implementation.

While the goal is always to meet the ideal standard, we acknowledge that decision-makers may be constrained by time during crises and may find it difficult to prescribe the proposed RST to modellers. However, we think the value of adopting such a tool to be greater than the additional workload it may create, by providing a mechanism to monitor the type and quality of evidence they receive, putting them in a commanding position to assess the level of confidence with which they can make decisions. Nonetheless, the RST provides flexibility to decision-makers and funders in countries with saturated local capacity (to provide and consume evidence) and rapidly evolving policy needs, by starting with the minimum standard of reporting at their initial engagement with modellers. Urgent decision needs, for e.g., closure of international borders may be aided by, at least, the minimum standard of reporting. For non-immediate decisions, for e.g., reopening of schools, an acceptable if not ideal reporting standard should be sought. We see the RST as a practical tool to deal with the constraints on all stakeholders during pandemics while re-emphasising the use of the highest possible quality of evidence.

The RST echoes the significance and benefits of a collaborative approach to modelling for policy. First, it allows the funders, and decision-makers to screen the modellers who have the calibre to deliver the required standard of reporting by demanding their commitment to gradually improve the standard to meet the ideal standard. This way, decision-makers can be assured that the evidence used in their decisions will be appropriate, adequate, and timely, which all sit at the heart of evidence-informed decision-making. Second, it can improve transparency of the process and increase accountability of all stakeholders involved. Funders and decision-makers can seek justification from modellers when they fail to improve or meet criteria in the RST, and the general public can demand that decision-makers and funders only engage with modellers who have committed to such RST and seek justification when they do not or consistently use sub-standard quality of evidence in their decisions. This way modellers are obliged to report their finding even when they contradict results of other modellers or recommend against a favourable action of a decision-maker. While the RST cannot be a binding constraint, they can certainly be used as tool to increase public ownership to such models, maintain accountability, and legitimise decisions.

However, adopting the RST is driven by policy urgency and local capacity of a country. These factors are subjective and differ by context. Hence, a wider stakeholder engagement is advised (explored below) to build consensus and make a value judgement on what is feasible and acceptable. This applies to both, choosing the appropriate reporting standard at the initial stage and the timelines to transition to a higher quality of reporting.

## 4 A participatory process in policy implementation

**Fig. 3:**
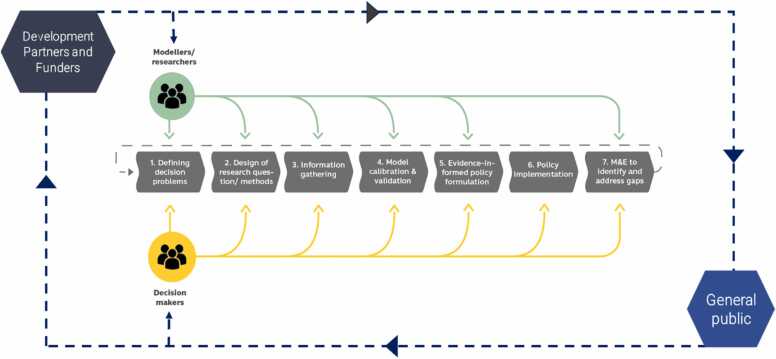
Collaborative modelling for effective policy implementation and evaluation.

We capitalise on an existing framework on the collaborative process between modellers and stakeholders (Behrend et al., 2020), to address challenges including transparency, inclusive decision-making, and accountability that hinder successful policy implementation (Rajan et al., 2020). This has been done in two ways: First, the addition of the fitness-for-purpose flowchart and the RST provide a practical guide and tangible avenues for decision-makers and funders to participate in modelling efforts. Second, by extending the definition of stakeholders to funders, researchers from other fields, ground-level implementers, and general public, depicted in Fig. 3, we define their roles and contributions in this process and allow each to be held accountable for their actions or the lack thereof. This process is iterative and should be applied to continuously monitor, evaluate, and revise decisions.

Funders can play a crucial role in this process by bringing in modellers with a reputation for maintaining professional standards to generate evidence on pressing decision problems. By seeking commitments from the decision-makers and modellers to adopt the fitness-for-purpose flowchart and the RST, funders can ensure adequate levels of collaboration required for an effective modelling to inform policy and reap the benefits discussed above. The uncertainty surrounding the virus and its interaction with society means continuous learning remains key to understanding and tackling the outbreak (Layne et al., 2020, Weible et al., 2020). Hence, this collaborative effort should extend to researchers from other fields, implementers, and general public who can provide society-based information such as nuanced local culture, values, behaviour, environment, and how different population groups are being affected by the epidemic (Layne et al., 2020). In LMICs, religious gatherings, intergenerational living, large informal sector with daily wage earners, and migrants living in congested areas, may prohibit the ability to maintain social distancing (Amanda Glassman KCaRS, 2020). Modelling outcomes and policies devoid of such context, for example, stringent lockdowns in densely populated and poor communities may result in unintended consequences like excess mortality (Lai et al., 2020), starvation, and poverty (Internaltional Labour Organization, 2020), or create new hot spots for the virus (Tan, 2020)*.* Furthermore, in light of yet another coronavirus disease and emerging climatic hazards, adopting the interdisciplinary One Health approach becomes crucial. By bringing experts from various fields, it can offer an understanding of the complex interrelationship between animals, humans, and the environment, and preventative interventions that can reduce the threat of outbreak such as bio-surveillance of live animal markets, identifying sources of pathogens, improved biosecurity, public education on zoonotic diseases, etc (El Zowalaty and Jarhult, 2020). A detailed guide from WHO on addressing zoonotic diseases using the one health approach is discussed in this book (World Health Organization, 2019).

Early engagement with implementers (at different levels of government, agencies, etc.) can harmonise efforts, help coordinate a swift response that pandemics demand, and relay ground level information where logistical challenges can appear while implementing a response (Sigurdsson et al., 2020). Hence, modelling process and results need to be integrated into the wider pandemic considerations and not viewed as a standalone activity. As depicted in Fig. 3, this can be done by bringing in other researchers who can monitor and evaluate the value of recommendations made by such models as well as find new areas for research. For example, conducting the cost-effectiveness of a particular intervention (for e.g., imposing a lockdown) (Appleby, 2020) by considering wider societal costs such as excess mortality, increased hunger poverty, impact on routine immunisation, increased mental health issues, national gross domestic product (GDP), etc. against the reduction in infection and deaths due to COIVD-19. A collaborative modelling effort can provide insights into such trade-offs which remains crucial for a sustainable response on our road to recovery from COVID-19.

Broadening the definition of stakeholders during pandemics can inform the value, feasibility, acceptability, and allow necessary fine-tuning of any policy intervention recommended by models (Ashton, 2020, Legido-Quigley et al., 2020). Such an inclusive and transparent process can bring a sense of public ownership to decisions and instil trust, increasing cooperation and adherence to policy measures from the public, which remains vital in fighting the COVID-19 pandemic. Leveraging decisions with such a process can help avoid public scrutiny and keep distant selected voices and vested interests (Norheim et al., 2020). It is important to note that such engagement is demanding on the time and capacity of researchers and decision-makers. For those reasons, it is important to establish this process early on to ensure appropriate planning and resourcing for this modality of engagement. This framework for stakeholder engagement can be applied to other types of research beyond disease modelling and was adopted while developing Thailand’s triage protocol to prioritise critical care resources during the first outbreak of COVID-19 which can offer actionable details of this framework (Marshall et al., 2021).

## 5 Conclusion

Pandemics like COVID-19 are a public health, social, economic, and national security issue, and forthcoming policy responses will touch all aspects of our daily lives. Thus, it is imperative that such policies are informed by rigorous and appropriate methods designed for local settings, are based on robust evidence, and are contested and enacted through public deliberation. This can instil trust and increase adherence to the policies by the public and can ultimately decide the trajectory of the epidemic (Udow-Phillips and Lantz, 2020; Devine, et al). Mathematical models will remain fundamental in informing policy responses to subsequent outbreaks, new variants of the virus, lifting border control measures, and impact evaluation of newly developed treatments and vaccines. Hence, we encourage the use of the decision tools offered in this paper to address the challenges in using models for policymaking which may continue to surface during this or future outbreaks. Finally, adoption of such tools can provide a strong justification for increased funding that is essential for preventing and responding to public health emergencies.

## CRediT authorship contribution statement

**YT, SKC, YL, SD, JK, and HC** together convened the engagement (online meetings) with policymakers, conducted the online surveys, and searched the literature. **SW, CL, GL, HS, MH, MB, SN, SS, DW, KC, MG, and RH** participated in the online meetings and surveys to share their experience in using evidence for models. **YT, SKC, and YL** wrote the first draft of the whole paper and revised the drafts. All other authors reviewed and contributed to the revision of the final draft.

## Funding

All organisations, authors, and members used their own funding for this work. The Health Intervention and Technology Assessment Program (HITAP) is a semi-autonomous research unit in the Ministry of Public Health, Thailand, and supports evidence-informed priority-setting and decision-making for healthcare. HITAP was supported by the International Decision Support Initiative (iDSI) and Program Manangement Unit-B (PMU-B) at the Ministry of Higher Education, Science, Research, and Innovation (MHESI), Royal Thai Government. iDSI is funded by the 10.13039/100000865Bill & Melinda Gates Foundation (OPP1202541), the UK’s Department for International Development, the 10.13039/100000877Rockefeller Foundation. HITAP is also supported by the Access and Delivery Partnership, which is hosted by the United Nations Development Programme and funded by the Government of Japan.

## Declaration of Competing Interest

The authors declare that they have no known competing financial interests or personal relationships that could have appeared to influence the work reported in this paper.

## Data Availability

The two online survey results can be made available upon request.
